# Mucosal Damage and Neutropenia Are Required for Candida albicans Dissemination 

**DOI:** 10.1371/journal.ppat.0040035

**Published:** 2008-02-15

**Authors:** Andrew Y Koh, Julia R Köhler, Kathleen T Coggshall, Nico Van Rooijen, Gerald B Pier

**Affiliations:** 1 Channing Laboratory, Department of Medicine, Brigham and Women's Hospital, Harvard Medical School, Boston, Massachusetts, United States of America; 2 Department of Medicine, Hematology/Oncology, Children's Hospital, Boston, Massachusetts, United States of America; 3 Department of Medicine, Infectious Diseases, Children's Hospital, Boston, Massachusetts, United States of America; 4 Department of Pediatric Oncology, Dana Farber Cancer Institute, Boston, Massachusetts, United States of America; 5 Department of Cell Biology and Immunology, Free University, Amsterdam, The Netherlands; University of California Los Angeles, United States of America

## Abstract

Candida albicans fungemia in cancer patients is thought to develop from initial gastrointestinal (GI) colonization with subsequent translocation into the bloodstream after administration of chemotherapy. It is unclear what components of the innate immune system are necessary for preventing C. albicans dissemination from the GI tract, but we have hypothesized that both neutropenia and GI mucosal damage are critical for allowing widespread invasive C. albicans disease. We investigated these parameters in a mouse model of C. albicans GI colonization that led to systemic spread after administration of immunosuppression and mucosal damage. After depleting resident GI intestinal flora with antibiotic treatment and achieving stable GI colonization levels of C. albicans, it was determined that systemic chemotherapy with cyclophosphamide led to 100% mortality, whereas selective neutrophil depletion, macrophage depletion, lymphopenia or GI mucosal disruption alone resulted in no mortality. Selective neutrophil depletion combined with GI mucosal disruption led to disseminated fungal infection and 100% mortality ensued. GI translocation and dissemination by C. albicans was also dependent on the organism's ability to transform from the yeast to the hyphal form. This mouse model of GI colonization and fungemia is useful for studying factors of innate host immunity needed to prevent invasive C. albicans disease as well as identifying virulence factors that are necessary for fungal GI colonization and dissemination. The model may also prove valuable for evaluating therapies to control C. albicans infections.

## Author Summary

Candida albicans is a fungus that lives harmlessly in the gastrointestinal (GI) tracts of humans. In cancer patients and patients undergoing bone marrow transplantation, however, the anti-cancer drugs that are administered to these patients also cause the undesired effect of suppressing the human immune system. The treatments allow C. albicans to spread into the blood and other organs and cause a severe disease. We found we could colonize the GI tracts of mice with C. albicans and then suppress the immune system with anti-cancer drugs to determine which components of the innate immune system (neutrophils, lymphocytes, macrophages, or GI tract integrity) are critical for preventing C. albicans from speading from the GI tract. We found that lowering the neutrophil counts and damaging the GI tract were both needed to cause systemic infection with C. albicans. We also found that the ability of C. albicans to switch from the yeast (spherical) form to the filamentous form is also important for establishing invasive disease. Our study provides new insights into the process of how a typically harmless microorganism inhabiting the GI tract can cause severe invasive disease once critical components of the host immune system are compromised.

## Introduction

Candida albicans is a ubiquitous commensal organism that can cause serious disseminated infections in cancer patients [1,2]. *Candida* spp*.* are the fourth leading cause of nosocomial bloodstream infections in the United States, with treatment costs estimated to be more than $2–$4 billion annually [3] and with attributable mortality rates estimated to be between 38% to 49% [4]. Among the various invasive fungal infections reported in cancer patients, candidiasis is the most common infection (58%–69%) [5–7], and over the past decade, the incidence of invasive fungal infections in this population has increased significantly [8].

The presumed mechanism for all invasive C. albicans disease involves initial mucosal surface colonization followed by invasion into the adjacent tissues and organs. In cancer patients, C. albicans usually colonizes the gastrointestinal (GI) tract with subsequent translocation into extraintestinal organs (i.e., mesenteric lymph nodes, blood stream, liver, and spleen) in the setting of chemotherapy-induced neutropenia and GI mucosal damage [9]. The three primary mechanisms that promote pathogenic microbial (bacterial and fungal) translocation in animal models are: 1) disruption of the normal GI microbiologic equilibrium allowing intestinal overgrowth of pathogens, 2) increased permeability of the intestinal mucosal barrier, and 3) deficiencies in the host immune defenses [10,11]. Not surprisingly, common risk factors for developing candidemia in human patients include neutropenia, mucositis, use of broad spectrum antibiotics, and invasive medical procedures [6,12].

The majority of murine models of disseminated candidiasis have employed the administration of a chemotherapeutic agent (e.g., cyclophosphamide) followed by the subsequent intravenous injection of C. albicans [13,14] or simply the intravenous injection of high inocula of C. albicans [15,16]. Therefore, a murine model that first establishes GI colonization followed by translocation and dissemination via disruptions of select components of the innate host defense would afford valuable opportunities for studying the details of C. albicans pathogenesis, as well as delineating the relative roles of the major immune compartments or the actual immune mechanisms responsible for killing and/or controlling translocating C. albicans [10].

To evaluate normal host factors that must be disrupted to allow for GI colonization, fungal dissemination and significant morbidity or mortality due to candidemia, we have developed a reproducible mouse model wherein C. albicans GI colonization is first established and subsequent fungal dissemination is achieved following induction of immunosuppression and disruption of mucosal integrity. Both neutropenia and GI mucosal damage appear to be necessary for fungal dissemination in this murine model. Finally, we evaluated C. albicans mutants that had varying abilities to switch between the yeast and the hyphal growth form of C. albicans and found changes in virulence associated with an inability to switch to the hyphal phase*,* indicating the utility of this animal model for studying different aspects of the pathogenic process of C. albicans in the setting of GI colonization and dissemination.

## Results

### GI Colonization by C. albicans

**Figure 1:**
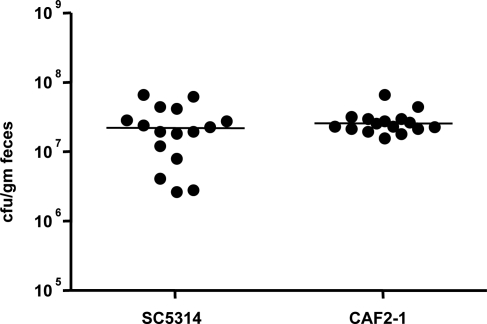
Gastrointestinal Colonization Levels of C3H/HeN Mice Fed C. albicans Strains SC5314 and CAF2–1 Mice were maintained on sterile water with 2 mg streptomycin/ml and 1,500 U penicillin G/ml throughout the duration of the experiment, and 0.2 mg gentamicin/ml was added once colonization was confirmed. Points represent results from individual animals, and horizontal lines represent the medians. *n* = 16 for each strain.

Wild-type C. albicans strains SC5314 (a strain that has been frequently used in various C. albicans murine models [17–20]) and CAF2–1 consistently colonized the mouse GI tract at comparable levels: SC5314 (median = 2.24 × 10^7^ cfu/g stool, first quartile = 1.07 × 10^7^, third quartile = 3.75 × 10^7^) and CAF2–1 (median = 2.60 × 10^7^ cfu/g stool, first quartile = 2.28 × 10^7^, third quartile = 3.19 × 10^7^; Figure 1). When tested up to 21 d later, there was no significant change in fungal colonization levels of the stool.

### Dissemination by C. albicans

Mice that were colonized with C. albicans strains SC5314 and CAF2–1, as well as non-*Candida* colonized control mice that were only decontaminated with antibiotics, were separated into 17 groups (8 mice/group, with the exception of 4 mice/group in Rag^−/−^ mice), treated with immunosuppressive regimens (Table 1), and the effect on dissemination of the different methods of immunosuppression determined (Table 2). Mice that were only decontaminated with antibiotics all survived after administration of immunosuppressive regimens—with the exception of 1 death in the group given mAb RB6-8C5 plus methotrexate (MTX) (Group 7). All groups that were colonized with C. albicans strains SC5314 and CAF2–1 then given immunosuppression showed levels of GI colonization comparable to those of mice not given immunosuppression (results shown in Figure 1; data for immunosuppressed mice not shown). Mice that were only given antibiotic decontamination and no immunosuppression after colonization with C. albicans (Group 1) also all survived.

**Table 1:**
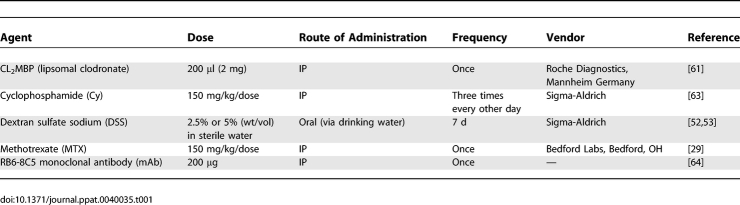
Immuosuppressive Regimens

**Table 2:**
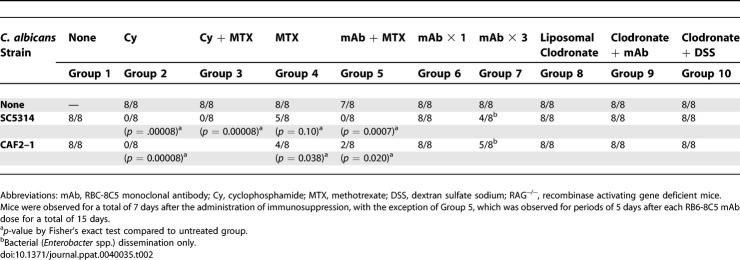
Survival of Mice after Administration of Immunosuppressive Regimens

**Table 2:**
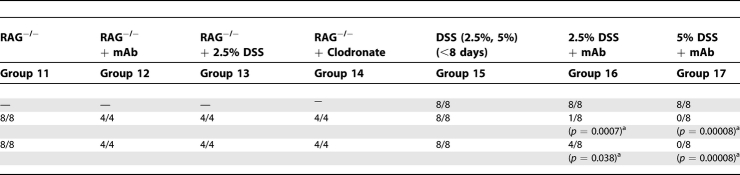
Extended.

#### Chemotherapy.

When either Cy alone (Group 2), Cy plus MTX (Group 3) or mAb RB6-8C5 plus MTX (Group 5) were administered, fungal dissemination occurred in all mice associated with a high level of mortality. Interestingly, when MTX alone (Group 4) was given, 3 of 8 mice in the group colonized with strain SC5314 and 4 of 8 mice in the group colonized with strain CAF2–1 died from disseminated disease. In contrast, when MTX was given concurrently with mAb RB6-8C5 (Group 5), all mice died following fungal dissemination.

#### Neutrophil depletion.

Mice that were made neutropenic after a single injection of 200 μg of anti-neutrophil mAb RB6-8C5 (mAb × 1) survived (Group 6). Even when given two additional doses of mAb RB6-8C5 (mAb × 3; Group 7) and thus made neutropenic for 15 d, there was no evidence of C. albicans dissemination in these mice. The four mice that died in this group only showed evidence of bacterial (e.g., Enterobacter spp.), not fungal, dissemination.

#### Macrophage depletion.

By immunohistochemical analysis, macrophages in both the liver and spleen were reduced after liposomal clodronate administration (Figure S1). By FACS analysis, F4/80 and CD11b double-stained macrophages were reduced in the spleen and bone marrow after liposomal clodronate administration from 26% to 16% (Figure S2). Mice given liposomal clodronate (Group 8), clodronate plus mAb RB6-8C5 (Group 9), or clodronate plus DSS (Group 10) all survived.

#### Lack of functional lymphocytes.

RAG^−/−^ mice colonized with strains SC5314 or CAF2–1 showed no evidence of dissemination (Group 11)—not even when mAb RB6-8C5 (Group 12), DSS (Group 13), or liposomal clodronate (Group 14) were subsequently administered to different groups of RAG^−/−^ mice.

#### GI mucosal disruption.

**Figure 2:**
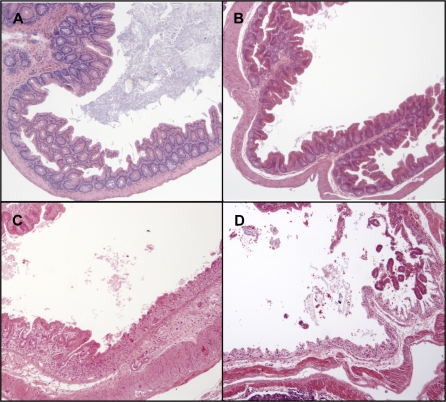
DSS-Induced Murine Gastrointestinal Mucosal Damage Histological sections of cecums from mice colonized with C. albicans strain SC5314 with continuation of penicillin, streptomycin, and gentamicin in the drinking water then given water with 2.5% DSS for various periods with or without induction of neutropenia. (A) No DSS, 200 μg of RB6-8C5 IP once. (B) DSS for 3 d. (C) DSS for 7 d. (D) DSS for 7 d and after the third day of DSS mice were given 200 μg of RB6-8C5 IP once and observed for 96 h after the RB6-8C5 dose. Magnification: Objective 10×. Sections in (A) and (B) have normal histologic appearance, whereas in panels (C) and (D) disruption of the epithelial barrier integrity was clearly seen.

There was no histologic evidence of any GI mucosal injury or damage in the ceca of mice given mAb RB6-8C5 or DSS for 3 d; in contrast, mice given DSS for 7 d had ulcerations and focal areas of inflammation noted in the cecum (Figure 2). The cecums of the mice that had received DSS for 7 d and one dose of mAb RB6-8C5 showed diffuse autolysis and sloughing. Mice given DSS alone showed no evidence of C. albicans dissemination (Group 15); however, when both DSS (2.5%) and mAb RB6-8C5 were given, disseminated disease and mortality was achieved (50% to 87.5% mortality). Increasing the DSS concentration from 2.5% (Group 16) to 5% (Group 17) while still giving a single 200 μg/mouse dose of mAb RB6-8C5 achieved 100% mortality associated with fungal dissemination to the liver.

**Figure 3:**
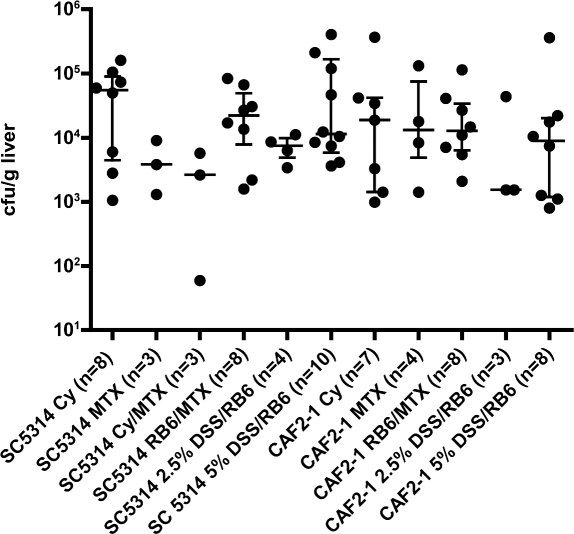
Viable Counts of C. albicans Strains SC5314 or CAF2–1 in the Livers of Deceased C3H/HeN Mice that Were Previously Colonized in the GI Tract with C. albicans and Subsequently Given Various Forms of Immunosuppression Cy, cyclophosphamide; MTX, methotrexate; RB6, RB6-8C5 mAb; DSS, dextran sulfate sodium. Points represent results from individual animals. Horizontal lines with bars represent median with interquartile range when *n* > 3, otherwise only the median is shown.

When mice were found moribund or died, the levels of C. albicans in the livers were comparable among animals given the different treatment regimens when both neutropenia and mucosal damage were induced (Figure 3). With the exception of the four mice noted previously in Group 7 (mAb × 3), there was no evidence of concurrent bacterial infection.

### Translocation Studies

We analyzed the effects of immunosuppression and/or mucosal damage treatments on translocation of C. albicans from the GI tract. All mice in these studies were colonized with C. albicans SC5314 and had levels of GI colonization comparable to that shown in Figure 1 (data not shown). Mice given no immunosuppression (Group A) all survived, and none appeared moribund and none grew C. albicans from the blood, spleen or MLN. Of note, fungi were found at very low levels in the livers of 4 (out of 20) healthy-appearing mice (9, 42, 208, and 369 cfu/g). The mice given mAb RB6-8C5 treatment only (Group B) all survived, and none appeared moribund. Similar to Group A, C. albicans was not found in the blood, spleen or MLN of mice only given the neutropenia-inducing mAb, but fungi were occasionally found in the liver (5 out of 20; median 9.88 × 10^2^ cfu/g, first quartile = 4.09 × 10^2^, third quartile = 2.68 × 10^3^); these are clearly higher levels than those found in the livers of mice in Group A which did not receive any immunosuppression. In contrast, 3 of the 4 mice from the group given neutropenia-inducing mAb RB6-8C5 plus MTX (Group C) died by day 4 post-immunosuppression, and 4 of 4 of these mice had died by day 5. All of these mice showed significant levels of C. albicans in the liver (median 8.77 × 10^3^ cfu/g, first quartile = 3.45 × 10^3^, third quartile = 2.51 × 10^4^). Finally, the mice given Cy only (Group D) all had C. albicans in the liver by days 4 and 5: (median 7.71 × 10^4^ cfu/g, first quartile = 3.87 × 10^4^, third quartile = 1.13 × 10^5^).

### C. albicans Morphogenesis as a Virulence Determinant

**Figure 4:**
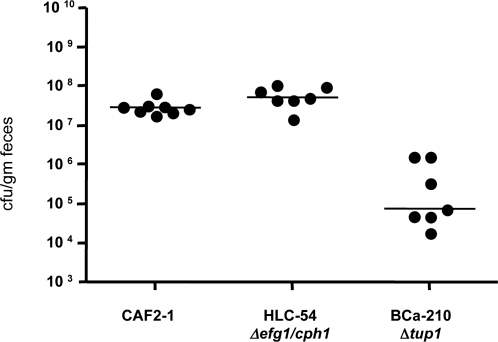
Gastrointestinal Colonization Levels of Female 6- to 8-Wk-Old C3H/HeN Mice Fed C. albicans Strains CAF2–1, HLC-54 (*Δefg1/cph1*), and BCa-210 (*Δtup1*) Points represent results from individual animals, and horizontal lines represent the medians. *n* = 8 for CAF2–1, *n* = 7 for *Δefg1/cph1* and *Δtup1*. Colonization levels with strain *Δtup1* were significantly lower (*p* = 0.0003 by Mann Whitney test) compared with other two strains.

**Figure 5:**
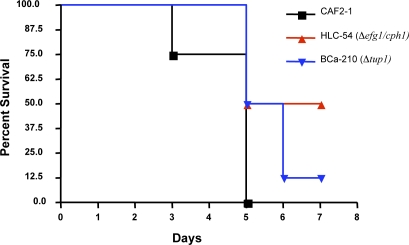
Survival Curve of C3H/HeN Mice Fed C. albicans Strains CAF2–1, HLC-54 (*Δefg1/cph1)*, or BCa-210 (Δ*tup1)* and Subsequently Given mAb RB6-8C5 (200 μg IP) and Methotrexate (150 mg/kg/dose IP) Median survival of mice colonized with strain *Δefg1/cph1* was significantly higher than that of mice colonized with wild-type C. albicans CAF2–1 (*p* = 0.01, log rank test). Each group contained 8 mice.

In order to assess the role of fungal morphogenesis as a virulence determinant in our murine model, we tested three C. albicans strains for their ability to colonize and disseminate following immunosuppression: strain CAF2–1 is a wild-type organism that was chosen because it, like the additional mutants we tested, only has one copy of the *URA3* gene [21]; strain HLC54 (*cph1/cph1 efg1/egf1* exhibits decreased filament formation, hereafter referred as Δ*efg1*/*cph1* [22]; and strain BCa-210 (*tup1/tup1*, hereafter referred to as Δ*tup1*) exhibits constitutive filamentous growth [23]. As shown in Figure 4, both CAF2–1 (median = 2.99 × 10^7^ cfu/g, first quartile = 2.38 × 10^7^, third quartile = 3.30 × 10^7^) and strain Δ*efg1/cph1* (median = 5.32 × 10^7^ cfu/g, first quartile = 4.69 × 10^7^, third quartile = 1.03 × 10^8^) were able to colonize the GI mucosa at comparable levels, while the Δ*tup1* strain colonized at a level 2-logs lower (median = 7.71 × 10^4^ cfu/g, first quartile = 5.0 × 10^4^, third quartile 1.66 × 10^6^, *p* = 0.0003 by Mann Whitney test compared with other two strains). This discrepancy is explained by the fact that the maximum concentration achievable of Δ*tup1* in water was approximately 2-logs lower than the levels for the wild-type and *Δefg1/cph1* strains—a difference which was consistent throughout the experiment. Interestingly, strain Δ*efg1/cph1* caused less mortality compared to wild-type C. albicans (8 of 16 mice given Δ*efg1/cph1* died versus 14 of 16 mice infected with wild-type, *p* = 0.02, Fisher's exact test), whereas in spite of the 2-log lower levels of strain Δ*tup1* in feces it caused mortality comparable to that of wild-type (7 of 8 mice died; Figure 5). In preliminary experiments with wild-type C. albicans strains SC5314 and CAF2–1, if we administered immunosuppression (e.g., mAb RB6-8C5 plus MTX) before final levels of GI colonization (between 10^7^ and 10^8^ cfu/g) had been established, dissemination was not induced, and all mice survived (data not shown). All of these surviving mice continued to be colonized with their respective strains of C. albicans at levels comparable to that achieved before administration of mAb and MTX.

Eight additional mice were administered higher concentrations of Δ*tup1* (5.5 × 10^5^ cfu/ml) in the drinking water, and GI colonization levels achieved with this higher dose were comparable to those of the wild-type C. albicans strain (median = 1.48 × 10^7^ cfu/g, first quartile = 5.20 × 10^6^, third quartile = 4.08 × 10^7^), indicating that the level of GI colonization can be increased for this strain. To determine if this constitutively filamentous strain could by itself induce mucosal damage sufficient to achieve dissemination, we administered the mice 200 μg of mAb RB6-8C5 only. No enhanced mucosal disruption sufficient to allow fungal dissemination in most mice was achieved by constitutive hyphal expression, as 7 of 8 colonized mice made only neutropenic still survived.

## Discussion

In this study, we attempted to devise a mouse model to study C. albicans pathogenesis and host factors leading to susceptibility to disseminated infection that emulate the pathophysiology that takes place in a human host, wherein receipt of broad-spectrum antibiotics, extensive hospitalization, or administration of immunosuppressive agents makes patients more vulnerable to invasive disease, often associated with intercurrent mucosal disruption owing to surgery, tumor invasion, or chemotherapy [24,25]. In neutropenic patients, the role of the gut as a source for disseminated candidiasis has been supported from autopsy studies [26].

Several murine models of GI-derived C. albicans fungemia and sepsis have been reported previously [27–32]. The levels of GI colonization we obtained were substantially higher than those achieved by prior investigators (1 to 3 log-fold higher) [31,33] and were maintained at least up to 21 d (our latest verification date). This higher colonization level is most likely due to the addition of penicillin to the drinking water. Penicillin most likely led to reductions of endogenous anaerobic bacterial flora, promoting subsequent intestinal overgrowth by C. albicans [34]. One other model did achieve comparable colonization levels by also administering adjunctive oral antibiotics [32]. The need for reduction of indigenous flora, particularly gram-negative bacteria, is critical given that bacteria are just as likely as the C. albicans to translocate and disseminate when immunosuppression is administered [35].

We were able to show this model recapitulates important aspects of human susceptibility to candidiasis, although there are some limitations to consider. The model was developed using adolescent/young adult 6- to 8-wk-old female C3H/HeN mice, and its applicability to other mouse strains or mice of other ages is not fully known. It would be difficult to use neonatal or infant mice in this model as others have done with acute C. albicans infections [28–30] due to the need for the animals to be able to drink both antibiotic water and fungi in the water and then give the immunosuppressive/mucosal disrupting agents, which would not be feasible with mice prior to weaning. C3H/HeN mice were used because of experience in a previous model of P. aeruginosa GI colonization and neutropenia-induced dissemination [35], and the fact that this strain has no major underlying immune deficit and is highly susceptible to DSS-induced colitis [36]. While we have not extensively studied other mouse strains, initial studies using Swiss Webster and C57/BL mice showed that comparable levels of GI colonization, mortality, and liver dissemination can be achieved when using Cy as the immunosuppressive/mucosal damaging agent (AYK and GBP, unpublished data). C. albicans administration via drinking water limits the ability to control the infecting dose as might be achieved by administering fungal cells by gavage. However, administering C. albicans in the drinking water for 5 d led to a reproducible and consistent GI colonization level (Figure 1) associated with dissemination following the disruption of specific host defenses. Additionally, we did not confirm the entire spectrum of bacterial decontamination achieved by the antibiotics, as we did not utilize anaerobic culture conditions or media specific for all types of aerobic and anaerobic flora. Therefore, we cannot be absolutely sure that we truly eradicated all indigenous aerobic and anaerobic flora. In any case, whatever residual aerobic or anaerobic flora that remained after antibiotic decontamination does not appear to have any pathogenic significance, as evidenced by the fact that mice given only antibiotic contamination and subsequent immunosuppression all appeared healthy and exhibited 0% mortality.

We also chose to measure fungal levels in livers as an indicator of dissemination. In other murine models of intravenous C. albicans infection fungal levels in the kidneys were used to confirm systemic infection was achieved. In preliminary experiments, we found that in mice that were colonized with C. albicans, given subsequent immunosuppression (cyclophosphamide or RB6-8C5 + MTX), and had no other detectable infectious cause of death (e.g. bacterial dissemination) that the liver (presence of C. albicans in 100% of livers from deceased mice) was a more reliable organ for confirmation of dissemination compared to the kidneys (presence of C. albicans in 50%–80% of kidneys in deceased mice; AYK and GBP, unpublished data). One other model using C. albicans GI colonization and chemotherapy-induced dissemination also noted 100% recovery from the livers but significantly less recovery from the kidneys [32]. Finally, for practical purposes we had to use organs for CFU enumeration from moribund then euthanized mice or mice that died between observations whose carcasses were frozen as close to the time of death as feasible. However, in limited studies we did compare the C. albicans yields from organs resected after freezing and storage with yields from organs resected from freshly euthanized mice and found the differences in CFU were not statistically different (AYK and GBP, unpublished data). Nonetheless, because there could be effects from post-mortem fungal growth or losses upon storage at −20°C one must be cautious in using levels of C. albicans in the organs as a measure of virulence.

We also attempted to determine how C. albicans might spread from the GI lumen to internal organs. Given the sporadic and low levels of hepatic dissemination in mice colonized with C. albicans but not receiving any immunosuppression, it is conceivable that C. albicans is able to translocate to the liver via the portal circulation or via the biliary tree in the absence of immunosuppression, but does not cause widespread disease because of a competent immune system that is able to prevent significant dissemination of the fungi. In our study, mice colonized with C. albicans and given only RB6-8C5 mAb also showed sporadic levels of hepatic dissemination, albeit at higher levels than in mice that did not receive immunosuppression. The higher fungal burden is most likely secondary to the lack of neutrophils and thus the subsequent diminished ability of the immunosuppressed animal to clear the C. albicans from the liver. But even in the absence of neutrophils, the fungal burden was not enough to cause frequent morbidity or death. Not surprisingly, when we administered systemic chemotherapeutic agents such as cyclophosphamide (resulting in both neutropenia and GI mucosal damage) we were able to reproducibly achieve widespread dissemination with *C. albicans.*

In the final analysis we found both neutropenia and disruption of the integrity of the GI mucosa were needed for fungal dissemination. In several intravenous murine models of C. albicans systemic infection, neutrophil depletion notably increases fungal burden and mortality [37,38]. In this murine model, however, neutropenia alone is not sufficient for extra-intestinal dissemination most likely due to the fact that C. albicans is still unable to breach the intact GI epithelium.

Although one prior study implicated the importance of CD4+ lymphocytes in protective immunity to systemic C. albicans infection [39], several studies in SCID mice have shown that a defect in the TH1 CD4+ T-cell response to C. albicans results in mucosal or esophageal candidiasis but not in systemic dissemination. Even in C5 deficient DBA/2 mice [38] and a recent murine HIV model [40], T cell depletion also does not result in disseminated C. albicans disease. Although the RB6-8C5 mAb at the dose used also induces some depletion of lymphocytes [35], we found that both C3H/HeN and RAG^−/−^ mice that lack mature lymphocytes were not susceptible to fungal disease following GI colonization and neutropenia alone. Thus, our murine model correlates with these previous studies: lymphopenia alone or in combination with macrophage depletion, neutrophil depletion, or GI mucosal disruption without neutropenia did not result in fungal dissemination or death of the colonized mouse.

Similarly, depleting macrophages only did not lead to any susceptibility to systemic fungal infection. Although murine peritoneal macrophages [41,42], pulmonary alveolar macrophages [43], Kuppfer's cells [44] and human peripheral blood monocytes [45] have the ability to phagocytose and kill C. albicans in vitro, in vivo studies on disseminated candidiasis have generated evidence both supporting [42,46–48] and refuting the importance [49–51] of macrophages. Of the two studies utilizing selective macrophage depletion [48,50], only one administered liposomal clodronate followed by intravenous fungal injection to produce a disseminated candidiasis. Spleens from the mice treated with clodronate lost their ability to trap yeast [48]. Furthermore, when macrophage-depleted mice were systemically challenged with C. albicans, not only was clearance of yeast decreased in blood, but kidneys had higher fungal burdens and overall mouse survival was decreased. Although we used the same dose of clodronate, we also utilized FACS analysis to quantify the macrophage depletion (37% depletion) rather than measuring peripheral blood monocyte counts (30%–85% depletion). Ultimately, our lack of finding a significant role for macrophages in host resistance to C. albicans may be related to the use of different models. In an intravenous model with direct inoculation, this degree of macrophage depletion may be enough of a deficit to lead to more severe disseminated disease. In our murine model, it may be that macrophages do play a role, but more substantial macrophage depletion may be necessary to see this effect.

Finally, while DSS has been used for experimental murine models of human inflammatory bowel disease [52,53], the histopathologic damage induced by DSS has some similarities to chemotherapy-induced mucositis in that both result in denudation and ulceration. Interestingly, when we maintained the same degree of neutropenia but increased the GI mucosal damage (by changing DSS from 2.5% to 5%), we notably increased the mortality to 100%, and this is most likely secondary to the increased mucosal damage caused by the higher concentration of DSS. These studies further support our hypothesis that it is a combination of neutropenia and GI mucosal damage that are critical for C. albicans dissemination in our murine model.

In the host, C. albicans grows as all known morphologic forms (budding yeast, pseudohyphal filaments, and true hyphae), and it has been postulated that the ability to induce hyphal formation is a critical virulence determinant [22]. Mutant strains of C. albicans that are incapable of hyphal formation have been found to be avirulent in murine models of disseminated candidiasis [54–56]. Whether it is simply that hyphae formation leads to an increased ability to invade host epithelial cells, or induces greater cytotoxicity, or whether the advantage of filamentous growth is in providing greater resistance to phagocytosis [22], the definitive mechanistic explanation linking hyphal formation to virulence is lacking. When testing the morphogenesis mutants, Δ*efg1/cph1*, which exhibit decreased filament formation [22], and *Δtup1*, which exhibits constitutive filamentous growth [23], we noted a nearly 2-log lower GI colonization level of the *Δtup1* mutant compared to the wild-type strain, CAF2–1. As noted earlier, this discrepancy in colonization level was most likely due to the difficulty of suspending *Δtup1* in drinking water and achieving higher concentrations. All of the mice colonized with strain *Δtup1* had colonization levels lower than the 10^7^ to 10^8^ cfu/g achieved with C. albicans strains SC5314 and CAF2–1, yet 7 of the 8 mice colonized with the *Δtup1* strain died following immunosuppression and GI mucosal damage. Since it is difficult to titrate inocula in our murine model, this finding of dissemination and mortality in the setting of significantly lower GI colonization levels may suggest that the *Δtup1* mutant is more virulent in this setting. The appropriate quantification of filamentous strains, however, is confounded by the fact that cells may not be properly separated, and thus CFUs are underestimated. In addition, filamentous strains such as *Δtup1* may adhere to the GI mucosa more tenaciously, and thus levels in the stool could be an underestimation of actual GI colonization levels. However, increased filamentous growth of the *Δtup1* mutant alone is not sufficient to disrupt the GI mucosa and allow for fungal dissemination even in the setting of neutropenia.

Our findings with CAF2–1 and Δ*efg1/cph1*were consistent with previous investigators that utilized an intravenous model of C. albicans dissemination [22,57,58]. However, in a model of hypoxic-induced GI translocation [59] and another study using oral inoculation followed by intraperitoneal (IP) injections of dexamethasone for immunosuppression [57], *Δefg1/cph1* was found to be more invasive compared to the wild-type CAF2–1. Whereas both of these studies used the presence of C. albicans in extraintestinal organs to define invasiveness or virulence, we used death attributable to C. albicans dissemination. Furthermore, we utilized a different means of immunosuppression and attempted to verify that bacterial translocation was not a confounding factor. Therefore, our findings with *Δefg1/cph1* support the hypothesis that the ability to form filaments is important for translocation and dissemination with *C. albicans.*

In conclusion, we have developed a murine model of C. albicans GI colonization following anti-microbial agent reductions in the indigenous flora and systemic spread during neutropenia that additionally requires GI mucosal damage. Neutropenia alone is not sufficient to produce disseminated C. albicans disease in this murine model. Being able to control these host factors should allow for a more detailed study of host and fungal factors needed to achieve GI colonization and systemic dissemination. These factors should thus be useful for evaluating pathogenesis as well as therapies to control C. albicans invasive infections.

## Materials and Methods

### Fungal strains and growth.

The strains of C. albicans used are listed in Table 3. C. albicans strains were grown overnight at 37°C in yeast extract-peptone-dextrose (YPD) broth, harvested by centrifugation, washed with PBS, and resuspended in PBS. C. albicans concentration was determined by use of a hemocytometer.

**Table 3:**
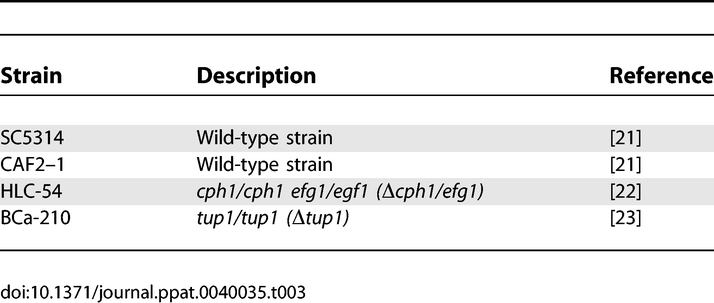
Fungal Strains Used in This Study

### Murine model of antibiotic-induced GI tract colonization by C. albicans and immunosuppressive-induced fungemia.

**Figure 6:**
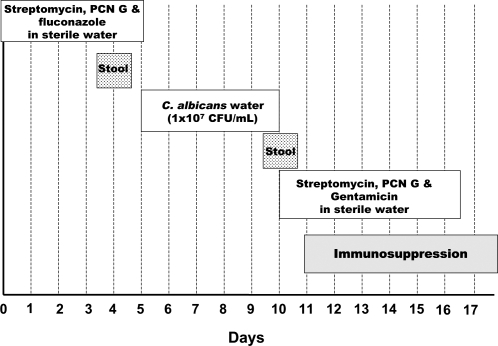
Schema for Murine Model of C. albicans Gastrointestinal Colonization and Dissemination after Administration of Immunosuppression

Six- to 8-wk-old female C3H/HeN mice (Harlan, http://www.harlan.com/models/c3h.asp) were housed as groups of 4 in sterilized cages equipped with filter hoods. In some experiments, 6-wk-old female recombinase activating gene deficient mice (Rag^−^/^−^, http://jaxmice.jax.org/strain/002216.html) were used. Mice were supplied with sterile bedding, sterile water and sterile mouse chow and maintained under specific pathogen-free conditions at the ARCM-MCP animal facility at Harvard Medical School in compliance with the Harvard Medical Area Institutional Animal Care and Use Committee guidelines. To deplete the indigenous GI bacterial and fungal flora, mice were fed sterile water with 2 mg streptomycin/ml (Research Product International, Mt. Prospect, IL), 1500 U penicillin G/ml (Sigma-Aldrich, St. Louis, MO), and 0.250 mg fluconazole/ml (Roxanne Laboratories, Columbus, OH) for 3 d, then switched to the same concentrations of streptomycin and penicillin G in their drinking water for one more day. Stool was collected from individual mice (0.030–0.050 g per stool pellet), homogenized in 1 ml 1% protease peptone, and 100 μl of the homogenate was spread and plated on yeast extract-peptone-dextrose (YPD), trypticase soy (TSA), or MacConkey agars to verify reduction of the targeted indigenous GI microbial flora. C. albicans strains (grown as described above) were added to sterile water with 2 mg streptomycin/ml, and 1,500 U penicillin G/ml at approximately 10^7^ cfu/ml, and then C. albicans was administered via the drinking water to mice for 5 d. Candidal levels were constant in the drinking water and therefore water bottles were not changed during this time. After 5 d of exposure to *Candida*, stool was again collected, homogenized in 1 ml 1% protease peptone, serially diluted and plated on YPD agar with 0.010 mg vancomycin/ml and 0.100 mg gentamicin/ml to measure levels of GI colonization by C. albicans. We then induced immunosuppression by treating the mice with the immunosuppressive agents listed in Table 1. After administration of immunosuppressive agents, mice were fed sterile water with 2 mg streptomycin/ml, 1,500 U penicillin G/ml, and 0.2 mg gentamicin/ml for the remainder of the experiment and monitored for morbidity for 7 d. Moribund mice were euthanized and along with mice found dead between observation periods, the carcasses were frozen at minus 20°C, later thawed, livers resected, homogenized in 1 ml 1% protease peptone, serially diluted and 100 μl of the homogenate was spread-plated on YPD with 0.100 mg gentamicin/ml (Research Product International, Mt. Prospect, IL) and 0.010 mg vancomycin/ml (Sigma-Aldrich), and additional 100 μl amounts plated onto TSA and MacConkey agar plates. Growth media were incubated at 37°C overnight under aerobic conditions. The presence of a homogeneous population of creamy-white colonies on YPD with gentamicin and vancomycin was used for confirmation of C. albicans systemic dissemination (Figure 6).

### Production or RB6-8C5 monoclonal antibody and induction of neutropenia.

The RB6-8C5 monoclonal antibody (mAb) specific for the Ly6 antigen highly expressed by polymorphonuclear neutrophils (PMN) was produced by growth of hybridoma cells in culture (Dulbecco's modified Eagle's medium with 10% fetal calf serum [FCS]) followed by purification of antibody by affinity chromatography, as previously described [35]*.* A single dose of 200 μg of RB6-8C5 was administered to C3H/HeN mice to produce a severe neutropenia (absolute neutrophil count < 100/mm^3^) for 5 d [35].

### In vivo macrophage depletion.

CL_2_MBP (clodronate) was a gift from Roche Diagnostics (Mannheim, Germany). Preparation of liposomes containing CL_2_MBP was done as described previously [60]. For assessment of macrophage depletion, three uninfected mice per group were inoculated IP with 200 μl of PBS liposomes or with CL_2_MBP liposomes (2 mg) [61]. At 2 d later, macrophages were quantified in the bone marrow and spleens using FACS analysis. Bone marrow and spleens were harvested from euthanized mice. To prepare single cell suspensions, spleens were sliced into small pieces with a scalpel, and the pieces placed at the ends of autoclaved frosted-glass microscope slides (Fisher Scientific, Pittsburgh, PA), previously immersed in Hank's balanced salt solution (HBSS; Gibco/Invitrogen, Carlsbad, CA) with 10% FCS. Splenic fragments were disrupted by opposing the slide ends and applying gentle pressure in round circular movements. The resulting splenic cell suspension was collected in a Petri dish and finally run over a 70 μm nylon cell strainer (BD Biosciences, San Diego, CA). For bone marrow cells, single cell suspensions were prepared by running bone marrow cells resuspended in HBSS with 10% FCS over a 70 μm nylon cell strainer. Cells from the three mice were pooled. Erythrocytes were eliminated using lysing solution (BD Biosciences, San Diego, CA). The remaining hematopoetic cells were washed and resuspended in PBS supplemented with 2% FCS, 1% bovine serum albumin, and 0.1% NaN_3_ (FACS buffer). Cells were blocked with Mouse Fc Block (BD Biosciences, San Diego, CA) at 1 μg per 10^6^ cells for 5 minutes at 4°C. Alexa-488-conjugated antibody to the mouse macrophage cell surface glycoprotein F4/80 (mAb BM8, Caltag/Invitrogen, Carlsbad, CA) and/or phycoerythrin-conjugated antibody to mouse CD11b (BD Biosciences, San Diego, CA) at 10 μg/ml/test were added to 1 × 10^6^ cells in 100 μl of FACS buffer then incubated at 4°C for 30 min followed by washing with FACS buffer. To determine background levels of fluorescence, non-antigen specific isotype controls (Alexa-488-conjugated-IgG_2a_ or phycoerythrin-conjugated-IgG_2b_ antibodies) were used as primary antibodies. After staining, cells were fixed with 1% paraformaldehyde. Fluorescence was detected on a Dako MoFlo High Performance Cell Sorter (Dako, Fort Collins, CO), and data analysis was performed using Summit Software v 4.3 (Dako, Fort Collins, CO). Gates were set to include CD11b and F4/80 double positive cells.

Livers and spleens from mice inoculated IP with 200 μl of PBS liposomes or CL_2_MBP liposomes (2 mg) 48 h prior to sacrifice were resected, fixed in Bouin's solution, and paraffin-embedded sections cut and mounted on slides. 5 μm sections of hepatic and splenic tissue were deparaffinized in xylene and rehydrated in a series of reverse ethanol washes (100%, 95%, and 80% ethanol sequentially). The samples were then blocked by incubation in histology blocking buffer (PBS containing 1% bovine serum albumin and 2% normal rat serum [Sigma-Aldrich]) for 15 min at 37°C. Samples were then incubated with 1 μg Alexa-488-conjugated antibody to the macrophage F4/80 antigen/ml for 90 min at 37°C. To detect background fluorescence, an appropriate isotype control antibody (1 μg rat IgG_2a_/ml) was used. The samples were then washed and sections visualized with the 10× alpha-plan lens on a Zeiss Axioplan 2 microscope, fitted with filter sets for GFP fluorescence, and a cooled CCD Hamamatsu Orca camera. Images were acquired and processed using MetaMorph (Molecular Devices Corporation, Sunnyvale, CA) and Adobe Photoshop software [62].

### In vivo gastrointestinal mucosal disruption.

To verify we had induced GI mucosal damage/disruption with dextran sodium sulfate (DSS), C3H/HeN mice (6- to 8-wk-old females) colonized with C. albicans strain SC5314 and maintained on sterile water with streptomycin, penicillin, and gentamicin to prevent bacterial recontamination were divided into four groups and sacrificed for cecal histology after the following specific treatments: Group A was given RB6-8C5 mAb once; Group B was given 5% DSS for 3 d; Group C was given 5% DSS for 7 d; and Group D was given 5% DSS water for 7 d, and after the third day of DSS water, mice were given mAb RB6-8C5 once, and observed for 96 h after the RB6-8C5 dose. Ceca were resected and immediately fixed in Bouin's solution. Sections were stained with hematoxylin and eosin and reviewed by a veterinary pathologist.

### Translocation studies.

To determine if the C. albicans colonizing the GI tract was translocating to extra-gastrointestinal sites, mice were colonized with C. albicans strain SC5314 using the protocol described above then organized into the following four groups containing 24 mice per group: Group A, no immunosuppression; Group B, neutropenia induced by IP injection of 200 μg RB6-8C5 mAb once; Group C, neutropenia and GI mucosal disruption induced by one IP injection of 200 μg RB6-8C5 mAb and a contemporary, single IP injection of 150 mg/kg MTX; and Group D, neutropenia and mucosal damage induced by three IP injections of 150 mg/kg Cy given every other day (Table 4). Four mice from each group were sacrificed on days 0, 1, 2, 3, 4, and 5 after immunosuppression was initiated. Blood (100 μl) was drawn from an ethanol-cleaned tail vein and spread-plated onto YPD agar with vancomycin and gentamicin, TSA, and MacConkey agar plates. Mice were then sacrificed. Mesenteric lymph nodes (MLN), spleens, and livers were immediately resected; organs were homogenized in 1 ml of 1% protease peptone; and 100 μl of homogenate was spread-plated on YPD agar with vancomycin and gentamicin, TSA, and MacConkey agar plates. The presence of a homogeneous population of creamy-white colonies on YPD with gentamicin and vancomycin were used for confirmation of the presence of C. albicans. Serial dilutions and plating for fungal enumeration were also done to obtain quantitative data (CFU per gram tissue and CFU per ml blood).

**Table 4:**
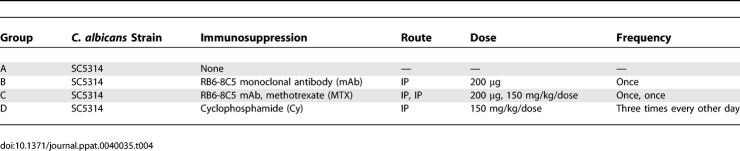
Translocation Study

### Statistical analyses.

Survival data were analyzed by Fisher's exact test and the survival curve was analyzed by the Kaplan-Meier log rank test using the GraphPad Prism software (San Diego, CA). Two-way comparisons of GI colonization levels were carried out using the Mann-Whitney tests and Prism software, and when multiple comparisons or more than two groups were analyzed, Bonferroni's correction to the significance level α was invoked.

## Supporting Information

Figure S1 Immunohistochemical Staining with Alexa-488 Anti-Mouse F4/80 of Murine Spleens and Liver after Liposomal Clodronate Administration (A) Isotype control (Alexa-488 rat IgG_2a_) staining of normal murine spleen.
(B) Alexa-488 rat anti-mouse F4/80 staining of normal murine spleen.
(C) Isotype control (Alexa-488 rat IgG_2a_) staining of normal murine spleen 48 h after IP clodronate.
(D) Alexa-488 rat anti-mouse F4/80 staining of murine spleen 48 h after IP clodronate.
(E) Isotype control (Alexa-488 rat IgG_2a_) staining of normal murine liver.
(F) Alexa-488 rat anti-mouse F4/80 staining of normal murine liver.
(G) Isotype control (Alexa-488 rat IgG_2a_) staining of normal murine liver 48 h after IP clodronate.
(H) Alexa-488 rat anti-mouse F4/80 staining of murine liver 48 h after IP clodronate. Magnification: Objective 10×.
(3.9 MB TIF)

Figure S2 Expression of F4/80 and CD11b on Murine (C3H/HeN) Bone Marrow and Spleen Cells Alexa-488–conjugated antibody to the mouse macrophage cell surface glycoprotein F4/80 and/or phycoerythrin-conjugated antibody to mouse CD11b were used. Cells from three mice were pooled for each analysis.
(A) Bone marrow from normal mice.
(B) Bone marrow from mice treated with liposomal clodronate 200 μl (2 mg) 48 h prior to analysis.
(C) Splenocytes from normal mice.
(D) Splenocytes from mice treated with liposomal clodronate 200 μl (2 mg) 48 h prior.
(4.9 MB TIF)

## References

[ppat-0040035-b001] DiNubile MJ, Hille D, Sable CA, Kartsonis NA (2005). Invasive candidiasis in cancer patients: Observations from a randomized clinical trial. J Infect.

[ppat-0040035-b002] Paulus SC, van Saene HK, Hemsworth S, Hughes J, Ng A (2005). A prospective study of septicaemia on a paediatric oncology unit: A three-year experience at The Royal Liverpool Children's Hospital, Alder Hey, UK. Eur J Cancer.

[ppat-0040035-b003] Wilson LS, Reyes CM, Stolpman M, Speckman J, Allen K (2002). The direct cost and incidence of systemic fungal infections. Value Health.

[ppat-0040035-b004] Gudlaugsson O, Gillespie S, Lee K, Vande Berg J, Hu J (2003). Attributable mortality of nosocomial candidemia, revisited. Clin Infect Dis.

[ppat-0040035-b005] Wang SM, Yang YJ, Chen JS, Lin HC, Chi CY (2005). Invasive fungal infections in pediatric patients with leukemia: emphasis on pulmonary and dermatological manifestations. Acta Paediatr Taiwan.

[ppat-0040035-b006] Rosen GP, Nielsen K, Glenn S, Abelson J, Deville J (2005). Invasive fungal infections in pediatric oncology patients: 11-year experience at a single institution. J Pediatr Hematol Oncol.

[ppat-0040035-b007] Bodey G, Bueltmann B, Duguid W, Gibbs D, Hanak H (1992). Fungal infections in cancer patients: An international autopsy survey. Eur J Clin Microbiol Infect Dis.

[ppat-0040035-b008] Groll AH, Shah PM, Mentzel C, Schneider M, Just-Nuebling G (1996). Trends in the postmortem epidemiology of invasive fungal infections at a university hospital. J Infect.

[ppat-0040035-b009] Pizzo PA, Poplack DG (2002). Principles and practice of pediatric oncology.

[ppat-0040035-b010] Berg RD (1999). Bacterial translocation from the gastrointestinal tract. Adv Exp Med Biol.

[ppat-0040035-b011] Shoham S, Levitz SM (2005). The immune response to fungal infections. Br J Haematol.

[ppat-0040035-b012] Pasqualotto AC, Nedel WL, Machado TS, Severo LC (2006). Risk factors and outcome for nosocomial breakthrough candidaemia. J Infect.

[ppat-0040035-b013] Andes D, Marchillo K, Stamstad T, Conklin R (2003). In vivo pharmacokinetics and pharmacodynamics of a new triazole, voriconazole, in a murine candidiasis model. Antimicrob Agents Chemother.

[ppat-0040035-b014] Coligan JE (2001). Current protocols in immunology.

[ppat-0040035-b015] Barnes JL, Osgood RW, Lee JC, King RD, Stein JH (1983). Host-parasite interactions in the pathogenesis of experimental renal candidiasis. Lab Invest.

[ppat-0040035-b016] Papadimitriou JM, Ashman RB (1986). The pathogenesis of acute systemic candidiasis in a susceptible inbred mouse strain. J Pathol.

[ppat-0040035-b017] Bader T, Schroppel K, Bentink S, Agabian N, Kohler G (2006). Role of calcineurin in stress resistance, morphogenesis, and virulence of a Candida albicans wild-type strain. Infect Immun.

[ppat-0040035-b018] Jackson BE, Mitchell BM, Wilhelmus KR (2007). Corneal virulence of Candida albicans strains deficient in Tup1-regulated genes. Invest Ophthalmol Vis Sci.

[ppat-0040035-b019] Pitarch A, Diez-Orejas R, Molero G, Pardo M, Sanchez M (2001). Analysis of the serologic response to systemic Candida albicans infection in a murine model. Proteomics.

[ppat-0040035-b020] Westwater C, Schofield DA, Nicholas PJ, Paulling EE, Balish E (2007). Candida glabrata and *Candida albicans;* dissimilar tissue tropism and infectivity in a gnotobiotic model of mucosal candidiasis. FEMS Immunol Med Microbiol.

[ppat-0040035-b021] Fonzi WA, Irwin MY (1993). Isogenic strain construction and gene mapping in Candida albicans. Genetics.

[ppat-0040035-b022] Lo HJ, Kohler JR, DiDomenico B, Loebenberg D, Cacciapuoti A (1997). Nonfilamentous C. albicans mutants are avirulent. Cell.

[ppat-0040035-b023] Braun BR, Johnson AD (1997). Control of filament formation in Candida albicans by the transcriptional repressor TUP1. Science.

[ppat-0040035-b024] Flynn PM, Marina NM, Rivera GK, Hughes WT (1993). Candida tropicalis infections in children with leukemia. Leuk Lymphoma.

[ppat-0040035-b025] Richet HM, Andremont A, Tancrede C, Pico JL, Jarvis WR (1991). Risk factors for candidemia in patients with acute lymphocytic leukemia. Rev Infect Dis.

[ppat-0040035-b026] Nucci M, Anaissie E (2001). Revisiting the source of candidemia: Skin or gut. Clin Infect Dis.

[ppat-0040035-b027] Mellado E, Cuenca-Estrella M, Regadera J, Gonzalez M, Diaz-Guerra TM (2000). Sustained gastrointestinal colonization and systemic dissemination by *Candida albicans, Candida tropicalis* and Candida parapsilosis in adult mice. Diagn Microbiol Infect Dis.

[ppat-0040035-b028] de Repentigny L, Phaneuf M, Mathieu LG (1992). Gastrointestinal colonization and systemic dissemination by Candida albicans and Candida tropicalis in intact and immunocompromised mice. Infect Immun.

[ppat-0040035-b029] Takahashi K, Kita E, Konishi M, Yoshimoto E, Mikasa K (2003). Translocation model of Candida albicans in DBA-2/J mice with protein calorie malnutrition mimics hematogenous candidiasis in humans. Microb Pathog.

[ppat-0040035-b030] Cole GT, Lynn KT, Seshan KR, Pope LM (1989). Gastrointestinal and systemic candidosis in immunocompromised mice. J Med Vet Mycol.

[ppat-0040035-b031] Wiesner SM, Jechorek RP, Garni RM, Bendel CM, Wells CL (2001). Gastrointestinal colonization by Candida albicans mutant strains in antibiotic-treated mice. Clin Diagn Lab Immunol.

[ppat-0040035-b032] Clemons KV, Gonzalez GM, Singh G, Imai J, Espiritu M (2006). Development of an orogastrointestinal mucosal model of candidiasis with dissemination to visceral organs. Antimicrob Agents Chemother.

[ppat-0040035-b033] Samonis G, Anaissie EJ, Rosenbaum B, Bodey GP (1990). A model of sustained gastrointestinal colonization by Candida albicans in healthy adult mice. Infect Immun.

[ppat-0040035-b034] Wells CL, Maddaus MA, Reynolds CM, Jechorek RP, Simmons RL (1987). Role of anaerobic flora in the translocation of aerobic and facultatively anaerobic intestinal bacteria. Infect Immun.

[ppat-0040035-b035] Koh AY, Priebe GP, Pier GB (2005). Virulence of Pseudomonas aeruginosa in a murine model of gastrointestinal colonization and dissemination in neutropenia. Infect Immun.

[ppat-0040035-b036] Mahler M, Bristol IJ, Leiter EH, Workman AE, Birkenmeier EH (1998). Differential susceptibility of inbred mouse strains to dextran sulfate sodium-induced colitis. Am J Physiol.

[ppat-0040035-b037] Fulurija A, Ashman RB, Papadimitriou JM (1996). Neutrophil depletion increases susceptibility to systemic and vaginal candidiasis in mice, and reveals differences between brain and kidney in mechanisms of host resistance. Microbiology.

[ppat-0040035-b038] Ashman RB, Papadimitriou JM, Fulurija A, Drysdale KE, Farah CS (2003). Role of complement C5 and T lymphocytes in pathogenesis of disseminated and mucosal candidiasis in susceptible DBA/2 mice. Microb Pathog.

[ppat-0040035-b039] Cenci E, Romani L, Vecchiarelli A, Puccetti P, Bistoni F (1989). Role of L3T4+ lymphocytes in protective immunity to systemic Candida albicans infection in mice. Infect Immun.

[ppat-0040035-b040] Marquis M, Lewandowski D, Dugas V, Aumont F, Senechal S (2006). CD8+ T cells but not polymorphonuclear leukocytes are required to limit chronic oral carriage of Candida albicans in transgenic mice expressing human immunodeficiency virus type 1. Infect Immun.

[ppat-0040035-b041] Cutler JE, Poor AH (1981). Effect of mouse phagocytes on Candida albicans in in vivo chambers. Infect Immun.

[ppat-0040035-b042] Redmond HP, Shou J, Gallagher HJ, Kelly CJ, Daly JM (1993). Macrophage-dependent candidacidal mechanisms in the murine system. Comparison of murine Kupffer cell and peritoneal macrophage candidacidal mechanisms. J Immunol.

[ppat-0040035-b043] Sawyer RT (1990). Experimental pulmonary candidiasis. Mycopathologia.

[ppat-0040035-b044] Sawyer RT, Garner RE, Hudson JA (1992). Effect of lectins on hepatic clearance and killing of Candida albicans by the isolated perfused mouse liver. Infect Immun.

[ppat-0040035-b045] Marodi L, Korchak HM, Johnston RB (1991). Mechanisms of host defense against *Candida* species. I. Phagocytosis by monocytes and monocyte-derived macrophages. J Immunol.

[ppat-0040035-b046] Baghian A, Lee KW (1988). Role of activated macrophages in resistance to systemic candidosis. J Leukoc Biol.

[ppat-0040035-b047] Bistoni F, Vecchiarelli A, Cenci E, Puccetti P, Marconi P (1986). Evidence for macrophage-mediated protection against lethal Candida albicans infection. Infect Immun.

[ppat-0040035-b048] Qian Q, Jutila MA, Van Rooijen N, Cutler JE (1994). Elimination of mouse splenic macrophages correlates with increased susceptibility to experimental disseminated candidiasis. J Immunol.

[ppat-0040035-b049] Garner RE, Kuruganti U, Czarniecki CW, Chiu HH, Domer JE (1989). In vivo immune responses to Candida albicans modified by treatment with recombinant murine gamma interferon. Infect Immun.

[ppat-0040035-b050] Lee KW, Balish E (1983). Systemic candidosis in silica-treated athymic and euthymic mice. Infect Immun.

[ppat-0040035-b051] Rogers TJ, Balish E (1977). The role of activated macrophages in resistance to experimental renal candidiasis. J Reticuloendothel Soc.

[ppat-0040035-b052] Cooper HS, Murthy SN, Shah RS, Sedergran DJ (1993). Clinicopathologic study of dextran sulfate sodium experimental murine colitis. Lab Invest.

[ppat-0040035-b053] Okayasu I, Hatakeyama S, Yamada M, Ohkusa T, Inagaki Y (1990). A novel method in the induction of reliable experimental acute and chronic ulcerative colitis in mice. Gastroenterology.

[ppat-0040035-b054] Fu Y, Ibrahim AS, Sheppard DC, Chen YC, French SW (2002). Candida albicans Als1p: An adhesin that is a downstream effector of the EFG1 filamentation pathway. Mol Microbiol.

[ppat-0040035-b055] Rocha CR, Schroppel K, Harcus D, Marcil A, Dignard D (2001). Signaling through adenylyl cyclase is essential for hyphal growth and virulence in the pathogenic fungus Candida albicans. Mol Biol Cell.

[ppat-0040035-b056] Marcil A, Harcus D, Thomas DY, Whiteway M (2002). Candida albicans killing by RAW 264.7 mouse macrophage cells: Effects of *Candida* genotype, infection ratios, and gamma interferon treatment. Infect Immun.

[ppat-0040035-b057] Bendel CM, Hess DJ, Garni RM, Henry-Stanley M, Wells CL (2003). Comparative virulence of Candida albicans yeast and filamentous forms in orally and intravenously inoculated mice. Crit Care Med.

[ppat-0040035-b058] Gale CA, Bendel CM, McClellan M, Hauser M, Becker JM (1998). Linkage of adhesion, filamentous growth, and virulence in Candida albicans to a single gene, *INT1*. Science.

[ppat-0040035-b059] Kim AS, Garni RM, Henry-Stanley MJ, Bendel CM, Erlandsen SL (2003). Hypoxia and extraintestinal dissemination of Candida albicans yeast forms. Shock.

[ppat-0040035-b060] Van Rooijen N, Sanders A (1994). Liposome mediated depletion of macrophages: Mechanism of action, preparation of liposomes and applications. J Immunol Methods.

[ppat-0040035-b061] Badell E, Oeuvray C, Moreno A, Soe S, van Rooijen N (2000). Human malaria in immunocompromised mice: An in vivo model to study defense mechanisms against Plasmodium falciparum. J Exp Med.

[ppat-0040035-b062] Lyczak JB, Pier GB (2002). Salmonella enterica serovar typhi modulates cell surface expression of its receptor, the cystic fibrosis transmembrane conductance regulator, on the intestinal epithelium. Infect Immun.

[ppat-0040035-b063] Cryz SJ, Furer E, Germanier R (1983). Passive protection against Pseudomonas aeruginosa infection in an experimental leukopenic mouse model. Infect Immun.

[ppat-0040035-b064] Czuprynski CJ, Brown JF, Wagner RD, Steinberg H (1994). Administration of antigranulocyte monoclonal antibody RB6-8C5 prevents expression of acquired resistance to Listeria monocytogenes infection in previously immunized mice. Infect Immun.

